# Probing the Interaction of Diester Internal Donors (ID) with AlEt_3_ on Ziegler-Natta Surfaces: A Comparison Between Binary (MgCl_2_/ID) and Ternary (MgCl_2_/ID/TiCl_4_) Formulations

**DOI:** 10.3390/molecules30102176

**Published:** 2025-05-15

**Authors:** Felicia Daniela Cannavacciuolo, Giuseppe Antinucci, Roberta Cipullo, Vincenzo Busico

**Affiliations:** Department of Chemical Sciences, Federico II University of Naples, via Cinthia, 80126 Napoli, Italy; feliciadaniela.cannavacciuolo@unina.it (F.D.C.); busico@unina.it (V.B.)

**Keywords:** Ziegler-Natta catalysts, polypropylene, internal donor, diester, catalyst productivity

## Abstract

Organic electron donors are essential components of Ziegler-Natta (ZN) catalysts to produce isotactic polypropylene. In particular, aromatic or aliphatic diesters are widely used as ‘Internal Donors’ (ID) in MgCl_2_/ID/TiCl_4_ precatalyst formulations. Diesters are reactive with AlEt_3_ (by far the most common ZN precatalyst activator) and are partly removed from the solid phase in the early stages of the polymerization process; this is detrimental for catalyst functioning, and a surrogate donor (‘External Donor’ (ED), usually an alkoxysilane) is added to the system to restore performance. Recent studies, however, demonstrated that even in cases where most of the diester is extracted by AlEt_3_, the active sites retain a ‘memory’ of it in several aspects of the catalytic behavior (such as, e.g., the average productivity and the polydispersity index of the polymer produced). Considering that the residual diester is always in molar excess with respect to the active Ti, one may speculate that long-lasting interactions between the latter and diester molecules can occur. In turn, this should imply that the reactivity of AlEt_3_ is different with binary MgCl_2_/ID or ternary MgCl_2_/ID/TiCl_4_ mixtures. In this work, the latter hypothesis was explored for a library of diester IDs with large structural diversity. In line with the anticipation, the fractional amount of ID extracted by AlEt_3_ was generally lower for ternary mixtures, although to an extent exquisitely dependent on diester structure.

## 1. Introduction

Ti-based Ziegler Natta (ZN) catalysts continue to dominate the industrial production of isotactic polypropylene (iPP) [1,2,3]. They consist of a solid precatalyst of composition MgCl_2_/Internal Donor (ID)/TiCl_4_, and a soluble AlR_3_/External Donor (ED) cocatalyst [1,2,3,4,5]. Catalyst performance, and most notably stereoselectivity, depends crucially on the nature of the ID/ED pair [6,7,8,9,10,11]. IDs that react with AlEt_3_, such as aromatic monoesters (e.g., benzoates), aromatic diesters (e.g., phthalates), and aliphatic diesters (e.g., succinates), are extracted from the solid catalyst in the early stages of the polymerization, although to variable extents [6,10,12,13]. In the case of monoesters, the process is extensive and associated with a dramatic loss of catalyst stereoselectivity, which must be restored by adding a proper ED (typically, another aromatic monoester [1,3,12,13]). With diester IDs, a qualitatively similar, albeit less extreme, picture has been reported, and alkoxysilanes are the most common EDs [7,8,10]. Some other IDs, instead, are unreactive with AlEt_3_ (like, e.g., 1,3-dimethoxypropanes), and adding an ED is not mandatory, although it can be beneficial for overall catalyst performance [5,10,14].

This scenario has been partly questioned by a recent comprehensive investigation of ZN catalysts making use of integrated High Throughput Experimentation (HTE) and Artificial Intelligence (AI) tools and methodologies [15]. In particular, the results indicated that diester IDs in the chemisorbed state are much less reactive with AlEt_3_ than monoester homologs [12,16,17,18], and that the residual ID, which is always in molar excess with respect to the active Ti [19,20,21,22] even in cases where a majority is extracted, leaves a durable imprint in the catalytic species. As a matter of fact, important catalyst properties, such as average productivity and polydispersity index of the produced iPP, are pre-determined by the ID and almost insensitive to the subsequently added ED. This points to a long-lasting association between diester molecules and active Ti.

Searching for some evidence of said association, in the present work we investigated comparatively the interaction of binary MgCl_2_/ID and ternary MgCl_2_/ID/TiCl_4_ mixtures with AlEt_3_. A library of diester IDs with large structural diversity was utilized. The full precatalysts were borrowed from the study of ref. [15], whereas MgCl_2_/ID mixtures were prepared by planetary ball milling. In the following sections, we report and discuss the experimental details and results of this study, including the implementation of a milling protocol ensuring the integrity of the ID molecules on the surface of the intensively activated MgCl_2_ particles.

## 2. Results and Discussion

**Figure 1:**
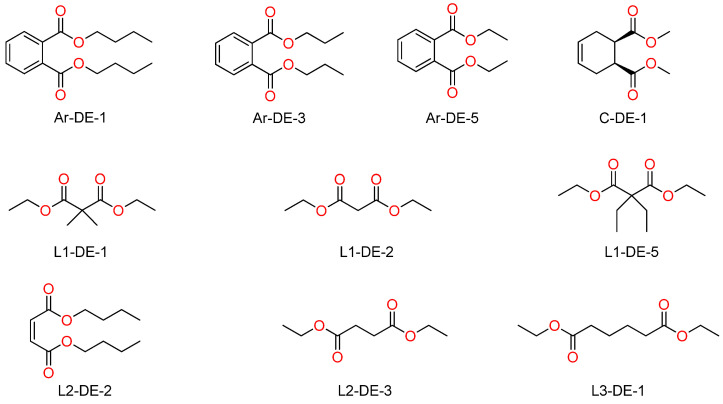
Structures and identification codes of the screened IDs.

A set of 10 diesters featuring variously substituted linear and cyclic aliphatic as well as aromatic structures (Figure 1) was selected for this work from a wider library of 22 diesters investigated in ref. [15] (for convenience, the ester identification codes in Figure 1 are the same used in the latter reference). Dibutylphthalate (Ar-DE-1) was chosen as a benchmark, due to its overwhelming importance in the iPP industry.

As noted above, the ternary MgCl_2_/ID/TiCl_4_ mixtures (full precatalysts) were already available from previous works [15,23]. The binary MgCl_2_/ID mixtures, instead, were purposely prepared by means of planetary ball milling [15,24]. This physical activation method is extremely efficient; indeed, it can be used to prepare metastable phases with nanosized particles [25,26,27]. For the present study, it has two important advantages: (a) it can be operated under a highly controlled inert atmosphere, thus hindering activated dry MgCl_2_ from water reuptake; and (b) it is much faster than chemical activation methods aimed at particle morphology control [1,3,28,29,30]; this is extremely important for catalyst application but unnecessary here. The main downside is the risk of diester thermal and/or mechanical degradation due to the high milling energy. Therefore, prior to systematic mixture preparations, four different grinding protocols were tested on the MgCl_2_/Ar-DE-1 system, namely a ‘dry’ (D) or a ‘wet’ (W) method, each applied in ‘harsh’ (h) or ‘mild’ (m) mode: the W method entailed the addition of an inert liquid diluent (dry heptane) into the jar to better dissipate the milling energy; the h and m application modes of each method, in turn, differed for the duration of the grinding intervals (20 min and 11 min, respectively) and of the pauses for reversing the direction of jar rotation (15 s and 240 s, respectively). For more details on hardware and protocols, see Section 3. All binary mixtures were characterized by means of powder X-ray diffraction (XRD) to estimate the average size of the MgCl_2_ coherence domains, and ^1^H NMR spectroscopy (after dissolution in methanol-*d*_4_) to measure the amount of strongly bound diester and the possible presence of a degraded diester fraction.

**Figure 2:**
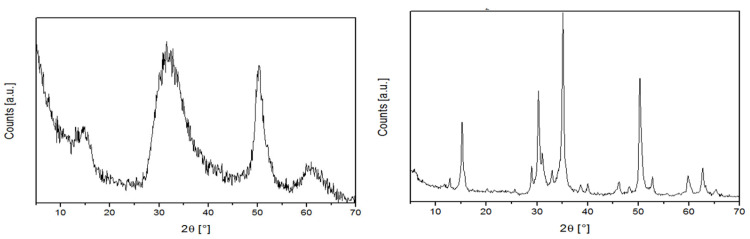
Powder XRD profiles (Cu K_α_ radiation) of MgCl_2_/Ar-DE-1 mixtures prepared by D (**left**) and W (**right**) planetary co-milling. For details see text.

Figure 2 reports the powder XRD profiles of representative MgCl_2_/Ar-DE-1 mixtures prepared with the D and W grinding method.

MgCl_2_ has a polymorphic crystal structure [31,32,33]. The common leitmotif of all modifications is the vertical stacking of structural layers, each consisting of two planes of close-packed Cl atoms with Mg atoms occupying all octahedral cavities in between. In the stable α modification, the succession of the Cl planes is (ABC)_n_, and the unit cell is hexagonal with axes *a* = 0.364 nm and *c* = 1.767 nm [31,32,33]. The *δ* form, instead, features rotational faults and irregular stacking of the Cl–Mg–Cl layers, resulting in nanosized crystallites characterized by lattice disorder [32,34,35,36]. Based on the XRD profiles, W-milled samples are in the α-form, whereas the very broad peaks of D-milled samples are indicative of the *δ*-MgCl_2_ form [34,35,36,37,38,39]. The average size of the coherence domains along the *a*-axis (<*L*_a_>) and the *c*-axis (<*L*_c_>) of the unit cell, i.e., perpendicularly and parallel to the basal planes of the structural layers, was calculated for all samples by applying the semi-empirical method of Allegra and Giunchi (using a customized Scherrer’s formula) [36]. The domains turned out to be very small (2–6 nm) in D-milled samples (1 and 2 of Table 1), and about 10-fold larger in W-milled ones (3 and 4 of Table 1). For both grinding methods, the choice of application mode turned out to be virtually inconsequential, especially with reference to <*L*_a_>.

**Figure 3:**
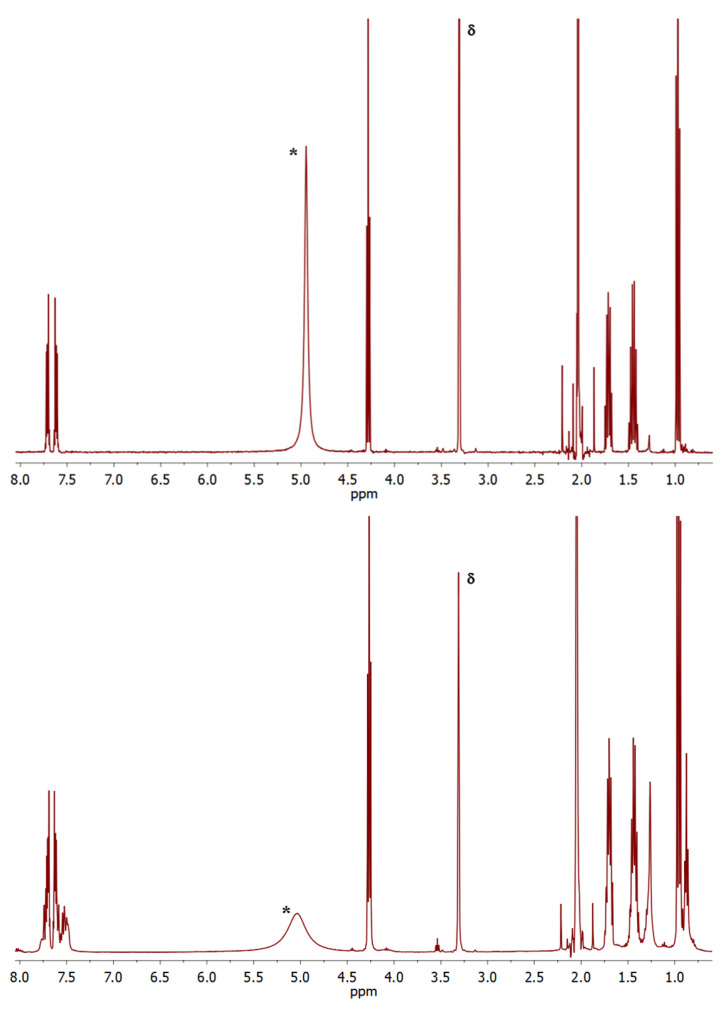
^1^H NMR spectra of Sample 1 (**top**) and Sample 4 (**bottom**) in Table 1. (*) = water; (δ) = non-deuterated methanol.

The results of the ^1^H NMR characterization of the samples are reported in the last two columns of Table 1; representative spectra are also shown in Figure 3. Notably, the weight amount of strongly bound diester (that is, residual to hot washing with heptane) was very similar for all four samples, notwithstanding the large differences in size of the XRD coherence domains. Considering that diester chemisorption occurs on the lateral terminations of the MgCl_2_ crystallites [40,41,42,43], our educated guess is that such terminations had similar average dimensions in all four samples, and that the much smaller coherence domains in D-milled ones should be ascribed primarily to the lattice disorder. Consistent with this assumption, the molar fraction of strongly bound diester is compatible with an average lateral dimension of the crystallites close to <*L*_a_> in the W-milled samples 3 and 4.

Importantly, the ^1^H NMR spectra of D-milled samples clearly indicated the presence of a non-negligible fraction of degraded diester (extra signals of aromatic protons in the range of *δ* = 7.3–7.6 ppm downfield of TMS, Figure 3-bottom [44]).

In view of the results just illustrated for the MgCl_2_/Ar-DE-1 mixtures, we decided to adopt the mW grinding protocol for preparing all binary MgCl_2_/ID mixtures for the present study. Table 2 lists the compositions of all binary MgCl_2_/ID and ternary MgCl_2_/ID/TiCl_4_ mixtures included in this study; in the latter, it is worthy to note that Ti and ID amounts were not far from equimolar.

Using a robotic reaction platform fully contained inside a glovebox (see Section 3 for details), all mixtures were suspended in a decane solution of AlEt_3_ ([Al]/[ID] = 25) at 80 °C under magnetic stirring; after 30 min, the solids were recovered by decantation, washed with two aliquots of heptane at 50 °C and one aliquot of pentane at 25 °C, and dried at 50 °C in a vacuum. Afterwards, the solids were dissolved in methanol-*d*_4_ and the residual ID content was measured by ^1^H NMR. The results are reported in Table 3.

In line with the hypothesis that prompted this work, the results in Table 3 indicate that the relative amount of diester ID residual to AlEt_3_ exposure in decane suspension was generally and appreciably lower in binary MgCl_2_/ID than in ternary MgCl_2_/ID/TiCl_4_ mixtures. The excess ID found in the latter (Δ(ID_res_)) was strongly dependent on the diester identity, ranging from about 20% for phthalates (namely, Ar-DE-1, Ar-DE-3, and Ar-DE-5) down to about 5% only for linear diesters lacking sterically demanding substituents (such as L1-DE-2, L2-DE-2, and L3-DE-1).

The relationship between Δ(ID_res_) and ID structure is all but trivial. An especially intriguing example is C-DE-1, with the lowest Δ(ID_res_) value (2%) in the set, notwithstanding a certain similarity in molecular shape with phthalates (but with an aliphatic nature and the presence of two chiral centers). Many different effects can contribute to this relation, because the steric and electronic features of the individual IDs likely impact on all processes in competition, including (but possibly not limited to) the chemisorption onto MgCl_2_, the formation of Lewis acid-base adducts with AlEt_3_, the subsequent reduction of the ester functionalities by AlEt_3_, and—last but not least—the postulated and still ill-defined association with the TiCl_n_ (n = 4 or 3) surface adducts. The very observation of a measurable Δ(ID_res_) (Table 3) confirms the hypothesis of such an association, which might consist in an adjacent positioning of the two moieties on the catalyst surface or even a direct bonding interaction; the latter, however, would require a nonepitaxial nature of (part of) the TiCl_n_ adsorbates [45].

**Figure 4:**
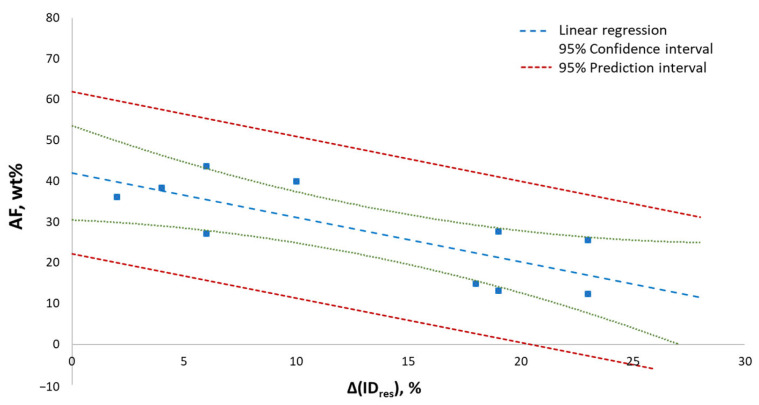
Correlation plot between Δ(ID_res_) and the stereoselectivity of catalyst systems based on the ternary mixtures of Table 2 and Table 3 in combination with AlEt_3_, expressed in terms of AF (see text). *R*^2^ = 0.60.

Intriguingly, a correlation plot between Δ(ID_res_) and the stereoselectivity of catalyst systems based on the ternary mixtures of Table 2 and Table 3 in combination with AlEt_3_, expressed in terms of the amorphous fraction (AF)—measured by analytical Crystallization Elution Fractionation (aCEF) [10,46,47,48]—of the produced iPP samples [15], highlighted an appreciable, albeit somewhat loose (*R*^2^~0.6), inverse correlation (Figure 4). In particular, catalyst systems modified with the three phthalates of Figure 1, with the highest values of Δ(ID_res_) in the investigated diester ID set, yielded iPP samples with AF values < 15 wt% even in the absence of an ED. We trace the higher stereoselectivity of ED-containing catalyst systems to the ‘lateral pressure’ exerted by the overall donor pool at high surface coverage, freezing the conformational motions of the donor molecules in the proximity of the catalytic pocket, and therefore enhancing their enantiodiscriminating ability due to the so-called ‘growing chain orientation mechanism of stereocontrol’ [49,50].

## 3. Materials and Methods

All manipulations of air-sensitive compounds were conducted under argon or nitrogen using Schlenk techniques and/or MBraun LabMaster 130 gloveboxes (Oberschleißheim, Germany). Hydrocarbon diluents (decane, heptane, and pentane HPLC-grade; Romil, Cambridgeshire, UK) were purified in an MBraun SPS-5 system by sequential passage through a fixed-bed column of A4 molecular sieves and a fixed-bed column of activated Cu catalyst (BASF, Ludwigshafen, Germany), so as to remove H_2_O and O_2_ to levels below 1 ppm_v_. AlEt_3_ was purchased from Chemtura Europe (Frauenfeld, Switzerland) and used as received. All diesters were purchased from TCI and used as received. Anhydrous MgCl_2_ was prepared according to protocols reported elsewhere [24].

Binary MgCl_2_/ID mixtures were prepared by means of a Retsch PM-100 planetary ball mill (Retsch GmbH, Haan, Germany), equipped with an airtight and chemically inert ceramic jar made with ZrO_2_ stabilized with Y [24]. Two different grinding methods, namely ‘Dry’ (D) or ‘Wet’ (W), each applied in a ‘harsh’ (h) or a ‘mild’ (m) mode, were tested. The W grinding method entails the presence of a diluent, namely anhydrous heptane. The two protocols had identical effective grinding time (6 h) and jar rotation velocity (650 rpm). In the h (m) application mode, grinding periods of 20 (11) min alternated with pauses of 15 (240) s for reversing the direction of jar rotation.

Operating inside a glovebox, aliquots of anhydrous MgCl_2_, diester ID (8.0 mol% with respect to Mg), and diluent (heptane) when appropriate, were loaded into the jar together with ≈87 g of grinding ceramic balls (3 mm diameter). The jar was then transferred to the mill. At the end of the milling process, the samples were collected in a glovebox. To separate any non-chemisorbed ID fraction, the recovered samples were washed twice with heptane aliquots at 50 °C and once with a pentane aliquot at 25 °C under magnetic stirring (600 rpm). Finally, the samples were dried at 50 °C under vacuum overnight.

All binary mixtures were characterized by powder X-ray diffraction (XRD) to identify the MgCl_2_ phase and measure the size of the coherence domains following a procedure described by Giunchi and Allegra [36]. XRD profiles were recorded using a Philips PW 1830 diffractometer (Philips, Eindhoven, The Netherland) equipped with a custom-made airtight cell with PVC windows, able to maintain a static atmosphere with negligible O_2_ and moisture contamination for at least 8 h. The cell was loaded in a glovebox and transferred to the diffractometer, where the diffraction was collected using Ni-filtered Cu K_α_ radiation (30 mA, 35 kV) with a step scanning procedure (2θ range between 5.0° and 70.0°, 0.10° step, 20 s counting time per step).

The amount of diester ID in each mixture was measured by means of quantitative ^1^H NMR spectroscopy, using a Bruker Avance DRX 400 spectrometer (Bruker Corporation, Eindhoven, The Netherland) operating at 400 MHz [10,51,52] with a 5 mm probe. The samples were dissolved in methanol-*d*_4_ (≈20 mg/mL), containing acetonitrile (1.0 μL) as an internal standard. The acquisition parameters were as follows: 90° pulse, 10 ppm spectral width, 60 s acquisition time, and 10 s relaxation delay. The resonance assignment was performed based on the ^1^H NMR spectra of the neat diester molecules recorded under the same conditions.

Full MgCl_2_/ID/TiCl_4_ precatalysts (ternary mixtures) were prepared in a previous collaborative investigation at the Japan Advance Institute of Science and Technology (JAIST). The full details of the synthesis and characterization have already been reported in Refs. [15,23,53].

Studies of ID extraction following exposure to AlEt_3_ in hydrocarbon suspension were carried out in a Freeslate Extended Core Module (XCM) robotic platform using established protocols [10,15,52]. In a typical experiment, an array of (up to 24) 8.0 mL glass vials, pretreated for at least 12 h at 200 °C under vacuum, were fitted with Parylene-coated magnetic mini stir-bars and placed into a 6 × 4 rack, which was then positioned in a deck bay. In each vial, a 25 ± 1 mg aliquot of MgCl_2_/ID or MgCl_2_/ID/TiCl_4_ mixture was suspended in dry decane (3.3 mL), and then 25 equivalents of AlEt_3_ (269–285 μmol) were added. The vials were capped, and the systems were allowed to react for 30 min at 80 °C under magnetic stirring (800 rpm). Then, stirring was stopped, and a cold quench (−30 °C) was performed by manually transferring the vials into a cold metal plate. At this point, the solid phase and the liquid phase of each vial were separated by centrifugation. The rack was placed in another deck bay, the vials were uncapped, and the supernatants were aspirated robotically (3.6 mL per vial). The solid phases, in turn, were robotically hot-washed twice with heptane and once with pentane, and finally were dried in a centrifugal evaporator for 10 h at 50 °C.

## 4. Conclusions

In the present work, we investigated how a variety of diester Internal Donors (ID), chemisorbed on activated MgCl_2_ in binary MgCl_2_/ID and ternary MgCl_2_/ID/TiCl_4_ mixtures, interacted with AlEt_3_ in hydrocarbon suspension under conditions mimicking the polymerization environment. The aim was to find experimental evidence of a lower reactivity of the diesters in ternary mixtures, suggesting a physical or chemical interaction with the TiCl_n_ surface species (n = 4 or 3) that durably imprints the catalytic behavior of the latter. The results did provide such evidence, although to an extent which is highly dependent on individual diester molecular structures (higher for phthalates, lower for aliphatic diesters lacking sterically demanding substituents). Overall, the picture emerging from this study suggests that the role of diester IDs in MgCl_2_-supported ZN catalysis to produce isotactic polypropylene has been somewhat underestimated, and that a major revision of the currently accepted models of catalytic species is likely justified.

## Figures and Tables

**Table 1:** Main results of the powder XRD and ^1^H NMR characterization of MgCl_2_/Ar-DE-1 mixtures prepared using various milling protocols. For details see text.

Sample	Grinding Protocol	<*L_c_*> (nm)	<*L_a_*> (nm)	*n*_Ar-DE-1,total_ (% wrt Mg)	*n*_Ar-DE-1,degraded_ (% wrt Mg)
1	Harsh-Dry (hD)	3.0	5.2	5.9	1.8
2	Mild-Dry (mD)	2.4	5.6	6.0	0.8
3	Harsh-Wet (hW)	32.1	26.3	5.6	Not detected
4	Mild-Wet (mW)	51.0	30.3	5.7	Not detected

**Table 2:** Composition of the investigated binary MgCl_2_/ID and ternary MgCl_2_/ID/TiCl_4_ mixtures.

	MgCl_2_/ID		MgCl_2_/ID/TiCl_4_				
ID	ID (wt%)	*n*_ID_ (% wrt Mg)	Ti (wt%)	*n*_Ti_ (% wrt Mg)	ID (wt%)	*n*_ID_ (% wrt Mg)	*n*(ID)/*n*(Ti)
Ar-DE-1	12.5	5.7	2.2	5.9	13.9	6.4	1.1
Ar-DE-3	11.8	6.0	1.7	4.5	12.4	6.3	1.4
Ar-DE-5	13.0	7.5	1.8	4.8	13.6	7.8	1.6
C-DE-1	9.8	6.5	2.4	6.4	8.2	5.3	0.8
L1-DE-1	7.2	4.9	2.0	5.3	9.6	6.5	1.2
L1-DE-2	10.1	7.8	2.4	6.4	12.6	10.0	1.6
L1-DE-5	9.2	5.5	2.7	7.2	10.2	6.0	0.8
L2-DE-2	8.9	6.0	2.1	5.6	15.9	10.8	1.9
L2-DE-3	9.1	6.8	2.5	6.7	7.6	5.6	0.8
L3-DE-1	11.7	7.9	1.8	4.8	13.5	9.2	1.9

**Table 3:** Relative amounts of residual ID in binary MgCl_2_/ID and ternary MgCl_2_/ID/TiCl_4_ mixtures after treatment with AlEt_3_ (30 min in decane suspension at 80 °C; see text).

ID	ID_res/binary_ (%)	ID_res/ternary_ (%)	Δ(ID_res_) ^(a)^ (%)	AF ^(b)^ (wt%)
Ar-DE-1	59	78	19	13.1
Ar-DE-3	72	90	18	14.8
Ar-DE-5	65	88	23	12.3
C-DE-1	68	70	2	36.1
L1-DE-1	62	81	19	27.7
L1-DE-2	76	80	4	38.4
L1-DE-5	67	90	23	25.5
L2-DE-2	54	60	6	43.6
L2-DE-3	64	74	10	40.0
L3-DE-1	59	65	6	27.1

^(a)^ Difference between the residual ID amounts of ternary MgCl_2_/ID/TiCl_4_ and binary MgCl_2_/ID mixtures; ^(b)^ amorphous fraction (AF) of the iPP produced in the presence of each ternary mixture (full precatalyst) in combination with AlEt_3_ (data from ref. [15]).

## Data Availability

The original contributions presented in the study are included in the article. Further inquiries can be directed at the corresponding authors.
